# Access to benznidazole for Chagas disease in the United States—Cautious optimism?

**DOI:** 10.1371/journal.pntd.0005794

**Published:** 2017-09-14

**Authors:** Jonathan D. Alpern, Rogelio Lopez-Velez, William M. Stauffer

**Affiliations:** 1 Division of Infectious Disease & International Medicine, Department of Internal Medicine, University of Minnesota, Minneapolis, Minnesota, United States of America; 2 National Referral Unit for Tropical Diseases, Infectious Diseases Department, Ramón y Cajal Hospital University Hospital, Madrid, Spain; Tulane University School of Public Health and Tropical Medicine, UNITED STATES

## Abstract

Drugs for neglected tropical diseases (NTD) are being excessively priced in the United States. Benznidazole, the first-line drug for Chagas disease, may become approved by the Food and Drug Administration (FDA) and manufactured by a private company in the US, thus placing it at risk of similar pricing. Chagas disease is an NTD caused by *Trypanosoma cruzi*; it is endemic to Latin America, infecting 8 million individuals. Human migration has changed the epidemiology causing nonendemic countries to face increased challenges in diagnosing and managing patients with Chagas disease. Only 2 drugs exist with proven efficacy: benznidazole and nifurtimox. Benznidazole has historically faced supply problems and drug shortages, limiting accessibility. In the US, it is currently only available under an investigational new drug (IND) protocol from the CDC and is provided free of charge to patients. However, 2 companies have stated that they intend to submit a New Drug Application (NDA) for FDA approval. Based on recent history of companies acquiring licensing rights for NTD drugs in the US with limited availability, it is likely that benznidazole will become excessively priced by the manufacturer—paradoxically making it less accessible. However, if the companies can be taken at their word, there may be reason for optimism.

## Background

Access to drugs for Neglected Tropical Diseases (NTD) is being threatened in the United States. Pharmaceutical companies have targeted licensure of these older drugs in order to corner specific markets—with the intent of raising prices for quick profit [1]. Such tactics have left economically disadvantaged patients unable to afford treatment. Benznidazole, the first-line treatment for Chagas disease, is currently provided at no cost under an Investigational New Drug (IND) protocol by the CDC but may soon become FDA-approved in the US. If this occurs, like other antiparasitic drugs with no competitor, it may paradoxically lead to dramatically increased pricing and less access for patients—as has recently occurred with albendazole and praziquantel.

The etiology of Chagas disease is the protozoan parasite *Trypanosoma cruzi*, causing an estimated 8 million infections and 10,000 deaths annually worldwide [2]. First described by Carlos Chagas in 1909, today it is considered one of the NTDs. *T*. *cruzi* is endemic to Latin America and is transmitted through the feces of the triatomine insect vector after a bite (primary mechanism), congenitally, by blood transfusion or organ donation, and via contaminated food or drink.

Although successful vector-control programs and mandatory blood bank screening have decreased its incidence considerably in Latin America since the 1990s, the epidemiology of Chagas disease has evolved due to human migration [3]. Whereas Chagas disease was historically considered a disease of the rural poor in Latin America, millions of migrants infected with *T*. *cruzi* have relocated to urban areas within Latin America and to nonendemic regions internationally. As Latin American migrant populations increase in nonendemic regions, the burden of Chagas disease has also increased. The US and Spain, with an estimated 300,000 and 42,000 migrants infected with *T*. *cruzi*, respectively, are countries that currently face public health, diagnosis, and treatment challenges [4]. In fact, a recent report estimates a 1.24% prevalence rate of Chagas disease among Latin American–born individuals in Los Angeles, which equates to >30,000 cases in Los Angeles alone [5].

Clinically, Chagas disease is divided into acute and chronic phases. The acute phase resolves spontaneously within 2–4 months. Without treatment, it becomes chronic and persists for the entirety of an individual’s lifetime. As many as 30% of infected people will develop chronic disease, most frequently involving the heart [6]. Because most migrants are likely exposed earlier in life, it is expected that complications such as cardiomyopathy will increasingly be observed in nonendemic areas as migrant populations age.

Only 2 drugs exist with proven efficacy in the treatment of Chagas disease: benznidazole and nifurtimox. Benznidazole is often considered first-line treatment due to a superior side-effect profile [6]. Treatment efficacy for benznidazole has been established for acute disease, reactivation in immunosuppressed hosts, congenital disease, and in children <15 years of age. However, there is controversy regarding the decision to “treat or not treat” in patients in the asymptomatic phase or with chronic Chagas cardiomyopathy. Most recently, the Benznidazole Evaluation for Interrupting Trypanosomiasis (BENEFIT) trial failed to show clinical benefit with benznidazole in patients with Chagas cardiomyopathy [7].

## History and global supply of benznidazole and nifurtimox

Historically, benznidazole has experienced significant supply chain disruptions and access problems. Benznidazole was introduced to market in 1971 by Roche as Rochagan or Radanil. In 2003, Roche transferred the rights of the drug to the public Brazilian State laboratory, Laboratorio Farmaceutico do Estado de Pernambuco (LAFEPE), which became the sole manufacturer under a directive of the Brazilian Ministry of Health [8]. In 2011, a shortage occurred in the face of increased demand due to improved recognition and screening efforts worldwide. The reason for the shortage was multifactorial. LAFEPE had recently transferred the responsibility of active pharmaceutical ingredient (API) production to a private company, Nortec Quimica, resulting in insufficient API production and a manufacturing delay [8]. In addition, LAFEPE lost the Good Manufacturing Practice (GMP) certificate, which was not renewed for over 3 years [9]. Multiple countries were affected by the shortage. Spain, for instance, lacked access to benznidazole for over a year and was forced to ration its supply for patients with acute infection, neonates, and immunosuppressed patients.

By 2012, production of a generic version of benznidazole, Abarax, was initiated in Argentina through a public and private joint initiative between the Ministry of Health and Mundo Sano Foundation. Maprimed, a local Argentine company, became responsible for API production, and ELEA, an Argentine pharmaceutical company, resumed the development and production of benznidazole. Although ELEA has guaranteed the production and distribution of Abarax throughout Latin America, the higher cost of Abarax has affected access for some patients [10]. Currently, ELEA and LAFEPE remain the only 2 sources of benznidazole.

By contrast, nifurtimox was originally released by Bayer as Lampit in 1967. After suspending production in 1997 due to lack of profitability, Bayer agreed to donate the drug to WHO in 2004 [11]. This donated drug has remained the primary source of nifurtimox worldwide. In the US, nifurtimox is not FDA approved but can be accessed through the CDC under the Expanded Access (Compassionate Use) protocol.

## Access to benznidazole in the US

In the US, benznidazole is only available from the CDC (LAFEPE product), which provides the drug free of charge through an investigational protocol. Yet, recent developments suggest that benznidazole may become available in the US as an FDA-approved product. Martin Shkreli, the hedge fund manager at Turing Pharmaceuticals notorious for hiking the price of Daraprim (pyrimethamine), acquired a majority stake in KaloBios, a private firm, in November 2015 and became CEO. KaloBios subsequently entered into an agreement to acquire the rights to benznidazole from Savant Neglected Diseases LLC [12]. While Shkreli was later fired by Turing Pharmaceuticals, KaloBios has continued to pursue FDA approval of benznidazole, recently submitting an Investigational New Drug (IND) application to the FDA. According to a KaloBios press release, they intend to submit a New Drug Application (NDA) in the first quarter of 2018, having received approval from the FDA to use efficacy and safety data performed previously [13].

KaloBios has recognized the value of a successful FDA approval for benznidazole. They are pursuing a Tropical Disease Priority Review Voucher (PRV) [12], an FDA-granted program intended to encourage the development of new drugs for certain tropical diseases. Private investors and pharmaceutical companies have learned that they can bring an existing drug to market with no development costs and receive a PRV. Rather than use the PRV as intended, these entities often sell the PRV, which are valued at hundreds of millions of dollars, [14] further increasing the profit they receive after increasing the price of the drug. KaloBios intends to submit benznidazole as a “new chemical entity,” which could result in up to 5 years of market exclusivity [12]. With no market competition, benznidazole is ripe for exorbitant pricing and at risk of joining the list of other unaffordable off-patent antiparasitic drugs in the US (Table 1).

**Table 1:** FDA-approved antiparasitic drugs.

FDA-Approved Antiparasitic Drugs	Antiparasitic Indication	Number of Listed Manufacturers***	Average Wholesale Price per Unit*
Daraprim (Pyrimethamine) 25 mg tablet **	Treatment of Toxoplasmosis, acute malaria; Malaria chemoprophylaxis	1	900
Impavido (Miltefosine) 50 mg capsule **	Treatment of visceral, cutaneous, and mucosal Leishmaniasis (certain species)	1	685.7
Albenza (albendazole) 200 mg tablet **	Treatment of Hydatid disease, Neurocysticercosis	1	201.27
Biltricide (praziquantel) 600 mg tablet**	Treatment of Schistosomiasis, Oriental liver flukes	1	99.64
Alinia (nitazoxanide)	Treatment of Giardiasis or Cryptosporidiasis		
500 mg tablet**		1	94.8
100 mg/5 ml suspension		1	6.45
Emverm (mebendazole) 100 mg chewable tablet**	Treatment of Enterobiasis (pinworm), Trichuriasis (whipworm), Ascaridiasis (common roundworm), Hookworms	1	442.8
Vermox (mebendazole) 500 mg chewable tablet	Treatment of Ascaridiasis (roundworm) and Trichuriasis (whipworm)	1	–
Coartem (Artemether/lumefantrine) 20 mg; 120 mg tablet	Treatment of uncomplicated Malaria	1	–
Lariam (mefloquine hydrochloride) 250 mg tablet	Treatment of uncomplicated Malaria; Malaria chemoprophylaxis	2	–
Humatin (paromomycin sulfate) 250 mg capsule	Treatment of Intestinal amebiasis	2	–
Tindamax (tinidazole) 250 mg tablet	Treatment of Trichomoniasis, giardiasis, intestinal amebiasis, bacterial vaginosis	5	–
Stromectol (ivermectin) 3 mg tablet	Treatment of intestinal strongyloidiasis, onchocerciasis	2	–
Malarone (atovaquone; proguanil hydrochloride) 62.5 mg; 25 mg tablet	Treatment of uncomplicated malaria; Malaria chemoprophylaxis	3	–
Aralen (chloroquine phosphate) 300 mg tablet	Treatment of uncomplicated nonfalciparum malaria; Malaria chemoprophyxis and extraintestinal Amaebiasis	4	–
Quinidine gluconate 80 mg/ml IV	Treatment of severe Malaria due to *Plasmodium falciparum*	1	–
Primaquine (primaquine phosphate) 15 mg tablet	Prevention of relapse of Malaria due to *P*. *vivax and P*. *ovale*	4	–
Amphotericin B (multiple IV formulations)	Treatment of visceral and mucosal Leishmaniasis	3	–
Qualaquin (quinine sulfate) 324 mg capsule	Treatment of uncomplicated malaria	6	–

* Data obtained from Truven Health Analytics (Red Book)

** High-cost drugs

*** Data obtained from the FDA's Orange Book

**Abbreviations**: FDA, Food and Drug Administration; IV, intravenously

## Reasons for cautious optimism?

However, there may be reasons for cautious optimism for affordable and dependable access to benznidazole. Through a strategic partnership initiated in February 2016 with the Drugs for Neglected Diseases *initiative* (DND*i*) and Mundo Sano Foundation, the pharmaceutical company Chemo Group (affiliated with ELEA) is also seeking FDA approval of benznidazole. This collaboration involves stakeholders who perform multidisciplinary Chagas research and have a history of working with disenfranchised populations that suffer from high rates NTDs in Latin America. This group of partners has pledged to make benznidazole affordable to patients throughout Latin America and in the US [15].

Meanwhile, KaloBios has also outlined an approach to pricing benznidazole fairly, pledging to use responsible and transparent pricing [12]. This unique pledge, while admirable, must be received with skepticism given recent events in NTD drug pricing in the US. However, if KaloBios follows through and is able to develop a business model that serves patients as well as their equity holders, they could serve as a role model for best practices in the pharmaceutical industry. Such a business model would demonstrate that a fair and transparent model can “do well, by doing good” as opposed to the “get rich quick, at the expense of human health” schemes we are currently experiencing.

We are left watching and waiting to see who obtains FDA approval, who follows through on public pledges, and if the burgeoning Latin American–born population with Chagas disease in the US will ultimately have access to treatment. Will we see a new and fair business model for drug pricing be pioneered for Chagas disease, or will the too often observed greed and “business as usual” practices continue to hold patients’ health hostage for the sake of excessive profits?
